# Supplementary material for: Perturbating Intramolecular Hydrogen Bonds through Substituent Effects or Non-Covalent Interactions
Source: Molecules. 2021 Jun 10;26(12):3556. doi: 10.3390/molecules26123556 (PMC8230504; doi:10.3390/molecules26123556)
Supplement: Supplementary file 1 [file molecules-26-03556-s001.zip › molecules-1256860-supplementary.pdf]

SUPPORTING INFORMATION

***

Perturbating Intramolecular hydrogen bonds through substituent effects or non-covalent interactions.

Al Mokhtar Lamsabhi, Otilia Mó and Manuel Yáñez

Departamento de Química (Módulo 13, Facultad de Ciencias) and Institute of Advanced Chemical Sciences (IadChem). Universidad Autónoma de Madrid. Campus de Excelencia UAM-CSIC. Cantoblanco, 28049-Madrid, Spain

E-mail: mokhtar.lamsabhi@uam.es, manuel.yanez@uam.es

CONTENTS

Figure S1. Relative stability of the $HOCH_2(CH_2)_nCH_2NH_2$ (n = 0-2) aminoalcohols stabilized by the formation of an O-H···N IMHB or a N-H···O IMHB.

Figure S2. Molecular structures of the substituted $HOCHX(CH_2)_nCH_2NH_2$ (n = 0-5, X = H, F, Cl, Br) aminoalcohols.

Figure S3. Molecular structures of the substituted $HOCH_2(CH_2)_nCHXNH_2$ (n = 0-5, X = F, Cl, Br) aminoalcohols.

Table S1. Second-order orbital interactions energies between the nitrogen lone-pair and the σ^*_{OH} antibonding orbital for the $HOCH_2(CH_2)_nCH_2NH_2$ (n = 0-3) amino-alcohols and their O···BeF_2 complexes

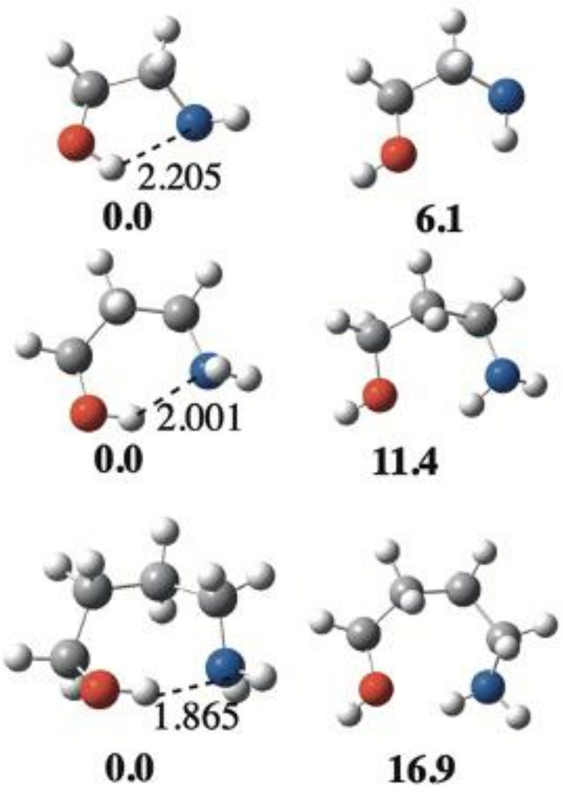

Figure S1. Relative stability of the $HOCH_2(CH_2)_nCH_2NH_2$ (n = 0-2) aminoalcohols stabilized by the formation of an O-H···N IMHB or a N-H···O IMHB.

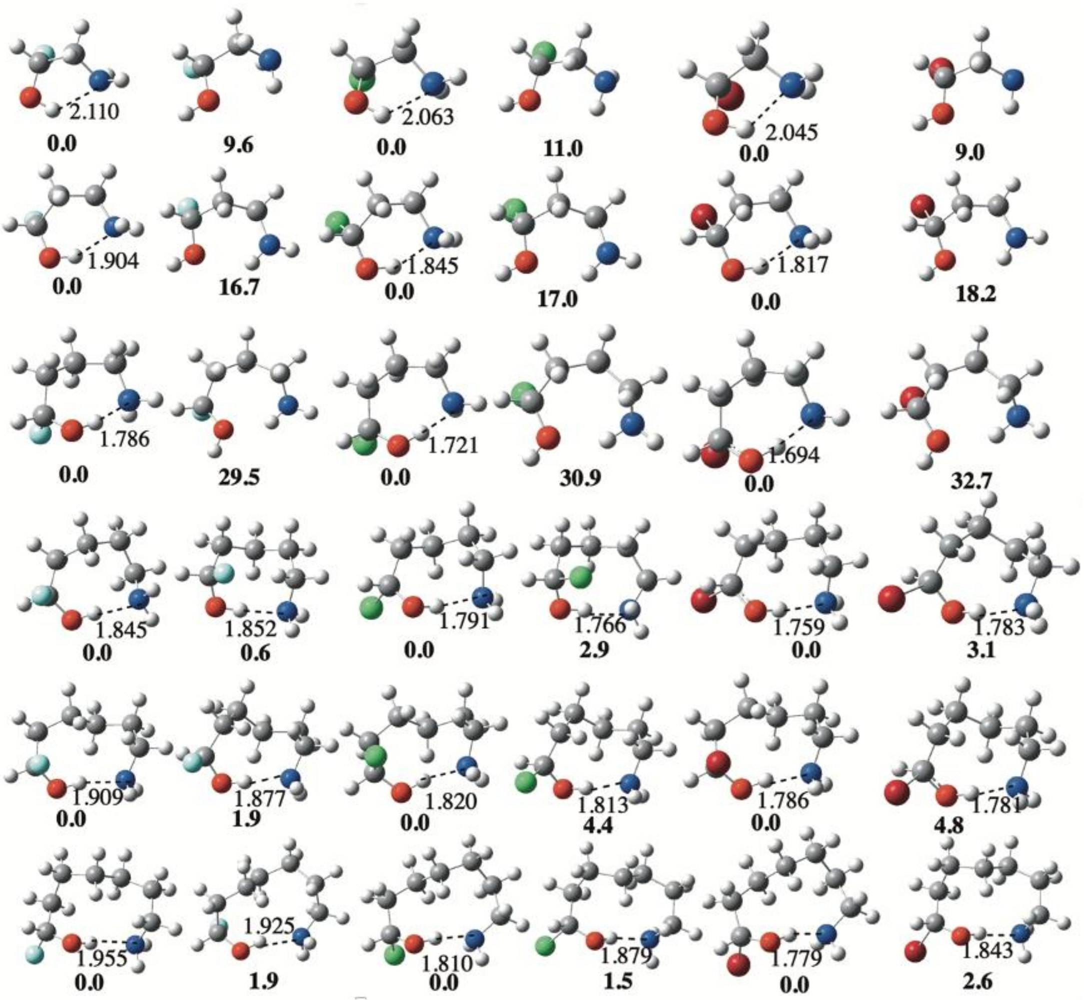

Figure S2. Molecular structures of the substituted $HOCHX(CH_2)_nCH_2NH_2$ (n = 0-5, X = H, F, Cl, Br) aminoalcohols, showing the bond-length, in Å, of the O-H···N (or NH···O) intramolecular hydrogen bonds. The relative energies ($kJ \cdot mol^{-1}$) with respect to the global minimum in each case is also given.

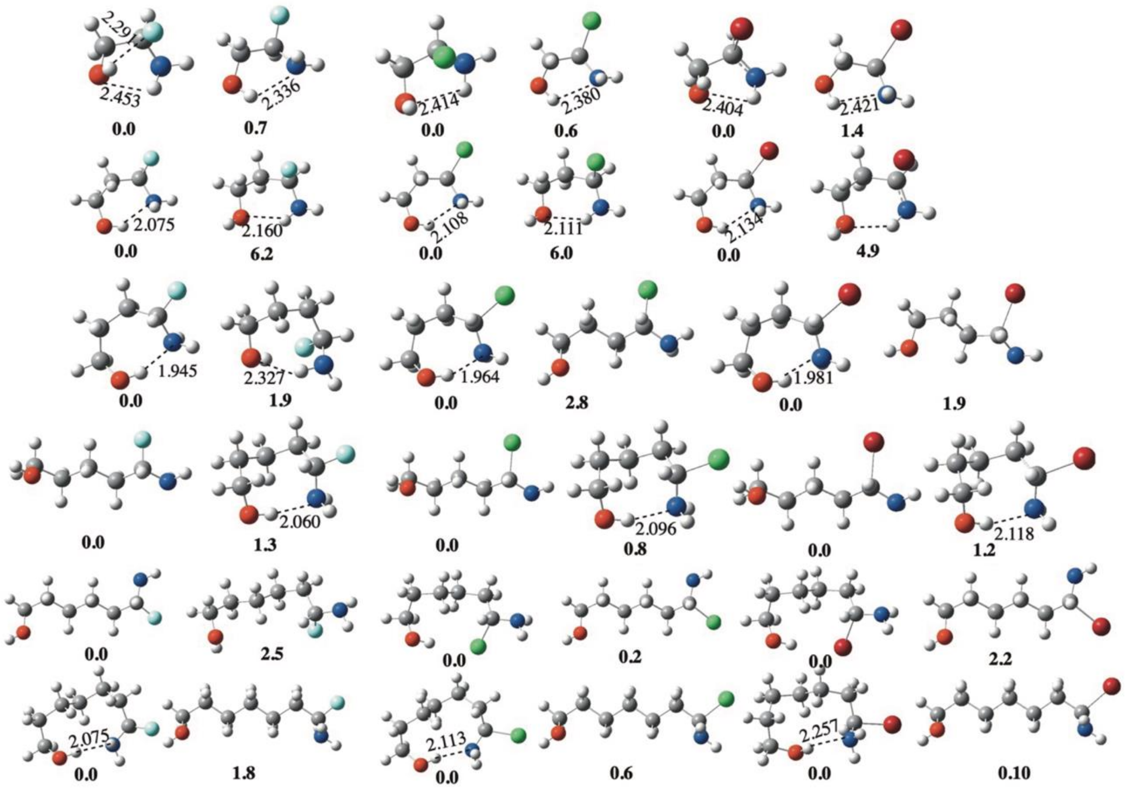

Figure S3. Molecular structures of the substituted $HOCH_2(CH_2)_nCHXNH_2$ (n = 0-5, X = F, Cl, Br) aminoalcohols, showing the bond-length, in Å, of the O-H···N (or NH···O) intramolecular hydrogen bonds. The relative energies ($kJ \cdot mol^{-1}$) with respect to the global minimum in each case is also given.

Table S1. Second-order orbital interactions energies ($kJ \cdot mol^{-1}$) between the nitrogen lone-pair and the σ^*_{OH} antibonding orbital for the $HOCH_2(CH_2)_nCH_2NH_2$ (n = 0-3) amino-alcohols and their O···BeF_2 complexes.

n	Isolated amino-alcohol	Complex with BeF2
0	6.8	24.8
1	32.3	112.1
2	67.6	215.0
3	49.2	203.9
